# Interplay of two low-barrier hydrogen bonds in long-distance proton-coupled electron transfer for water oxidation

**DOI:** 10.1093/pnasnexus/pgad423

**Published:** 2023-12-07

**Authors:** Keisuke Saito, Shunya Nishio, Hiroshi Ishikita

**Affiliations:** Department of Applied Chemistry, The University of Tokyo, Bunkyo-ku, Tokyo 113-8654, Japan; Research Center for Advanced Science and Technology, The University of Tokyo, Meguro-ku, Tokyo 153-8904, Japan; Department of Applied Chemistry, The University of Tokyo, Bunkyo-ku, Tokyo 113-8654, Japan; Department of Applied Chemistry, The University of Tokyo, Bunkyo-ku, Tokyo 113-8654, Japan; Research Center for Advanced Science and Technology, The University of Tokyo, Meguro-ku, Tokyo 153-8904, Japan

## Abstract

D1-Tyr161 (TyrZ) forms a low-barrier H-bond with D1-His190 and functions as a redox-active group in photosystem II. When oxidized to the radical form (TyrZ-O^•^), it accepts an electron from the oxygen-evolving Mn_4_CaO_5_ cluster, facilitating an increase in the oxidation state (S*_n_*; *n* = 0–3). In this study, we investigated the mechanism of how TyrZ-O^•^ drives proton-coupled electron transfer during the S_2_ to S_3_ transition using a quantum mechanical/molecular mechanical approach. In response to TyrZ-O^•^ formation and subsequent loss of the low-barrier H-bond, the ligand water molecule at the Ca^2+^ site (W4) reorients away from TyrZ and donates an H-bond to D1-Glu189 at Mn4 of Mn_4_CaO_5_ together with an adjacent water molecule. The H-bond donation to the Mn_4_CaO_5_ cluster triggers the release of the proton from the lowest p*K*_a_ site (W1 at Mn4) along the W1…D1-Asp61 low-barrier H-bond, leading to protonation of D1-Asp61. The interplay of the two low-barrier H-bonds, involving the Ca^2+^ interface and forming the extended Grotthuss-like network [TyrZ…D1-His190]-[Mn_4_CaO_5_]-[W1…D1-Asp61], rather than the direct electrostatic interaction, is likely a basis of the apparent long-distance interaction (11.4 Å) between TyrZ-O^•^ formation and D1-Asp61 protonation.

**Significance Statement**

Water oxidation occurs at the Mn_4_CaO_5_ cluster in photosystem II. The electron acceptor is the neutral radical form of the redox-active tyrosine, D1-Tyr161 (TyrZ-O^•^). The quantum mechanical/molecular mechanical calculations presented in this study indicate that TyrZ-O^•^ not only serves as an electron acceptor but also triggers protonation of D1-Asp61 in the second-flash induced transition (S_2_ to S_3_) by rearranging the H-bond pattern at the TyrZ…Mn_4_CaO_5_ interface upon the TyrZ-OH to TyrZ-O^•^ conversion and decreasing the lowest p*K*_a_ site of the Mn_4_CaO_5_ cluster, namely, a ligand water molecule (W1) at the dangling Mn site. The trigger works, as two low-barrier H-bonds already exist: one between TyrZ and D1-His190 and the other between W1 and D1-Asp61. This is the origin of the apparent long-distance (11.4 Å) interaction between TyrZ-O^•^ and D1-Asp61.

## Introduction

O_2_ evolution occurs at the oxygen-evolving Mn_4_CaO_5_ cluster in photosystem II (PSII) during the photocycle. The Mn_4_CaO_5_ cluster consists of the cubane region (Mn1, Mn2, Mn3, and Ca) and the dangling site (Mn4) (1, 2). In response to the electronic excitation of the accessory chlorophyll, an electron is transferred via pheophytin, the primary quinone to the secondary quinone, while the electronic hole is eventually stabilized at the chlorophyll pair (P_D1_ and P_D2_) as [P_D1_P_D2_]^•+^ (≍P_D1_^•+^ 3–6). P_D1_^•+^ ultimately abstracts an electron from the Mn_4_CaO_5_ cluster via redox-active D1-Tyr161 (TyrZ), and the oxidation state of the oxygen-evolving complex, S*_n_* (*n* = 0, 1, 2, and 3), increases (7). The number of protons released during the S-state transition is 1 for S_0_ to S_1_, 0 for S_1_ to S_2_, 1 for S_2_ to S_3_, and 2 for S_3_ to S_0_. Molecular oxygen (O_2_) formation occurs in the S_3_ to S_0_ transition. In the high oxidation state model, the Mn valence state in S_1_ is Mn(III)_2_Mn(IV)_2_, while in the low oxidation model, it is Mn(III)_4_ (8). Unless otherwise indicated, this study primarily focuses on S-states of the high oxidation state model (9). Since Mn2 and Mn3 reach the Mn(IV) state in S_1_ (6), either Mn1(III) or Mn4(III) serves as the oxidation site during the S_1_ to S_2_ transition. The oxidation of Mn1(III) to Mn1(IV) results in a short Mn1(IV)…O5 distance and a long Mn4(III)…O5 distance, forming the closed-cubane S_2_ conformation. Conversely, the oxidation of Mn4(III) to Mn4(IV) results in a long Mn1(III)…O5 distance and a short Mn4(IV)…O5 distance, forming the open-cubane S_2_ conformation (10, 11). The X-ray free electron laser (XFEL) structures identified the open-cubane S_2_ conformation, while the closed-cubane S_2_ conformation was not identified (12–14), possibly due to the greater energetic stability of the open-cubane conformation (12–16).

The release of 1 H^+^ is commonly observed in the S_0_ to S_1_ and S_2_ to S_3_ transitions. In the S_0_ to S_1_ transition, the protonated O4 site near Mn4 releases the proton along a chain of water molecules (O4–water chain) (17–19). The O4–water chain exists as a proton-conducting wire (in the original geometry of the crystal structure 1). Thus, it can mediate proton transfer by transforming the preproton transfer (pre-PT) H-bond pattern into the postproton transfer (post-PT) H-bond pattern, serving as a Grotthuss-like proton transfer pathway. For proton transfer, the formation of the proton-conducting wire is a prerequisite and an activation process (20). The preorganized H-bond network of the O4–water chain is consistent with a small H/D kinetic isotope effect (KIE) of 1.2 for the S_0_ to S_1_ transition observed using time-resolved infrared (TRIR) spectroscopy (19). Consistent with the experimental observations, quantum mechanical/molecular mechanical (QM/MM) calculations show that the release of the proton from protonated O4 was observed along a low-barrier H-bond in response to the oxidation of S_0_ to S_1_ (17). The result suggests that the process does not require protein dynamics (i.e. without conducting molecular dynamic simulations).

The S_2_ to S_3_ transition proceeds via two steps (19, 21, 22). A small KIE value of 1.2 was observed at the first step (19), which suggests that it proceeds in the preorganized H-bond network. The release of the proton toward the lumenal bulk region is observed in the second step with a slightly larger KIE value of 1.9 (19). The proton transfer pathway involves D1-Asp61, the second sphere ligand residue of the Mn_4_CaO_5_ cluster (23–30). The difference in the KIE value aligns with the activation process of the proton transfer. For the first step, QM/MM calculations show that a low-barrier H-bond forms between the ligand water molecule W1 and D1-Asp61 (26). In contrast, for the second step, the H-bond network, proceeding from D1-Asp61 toward D1-Glu65/D2-Glu312, forms only after H-bond network rearrangement during molecular dynamics (MD) simulations (26, 29).

The presence of a low-barrier H-bond is a prerequisite for efficient proton transfer (31). The low-barrier H-bond forms only after the oxidation of the Mn_4_CaO_5_ cluster in the S_0_ to S_1_ transition (17, 18), whereas it already exists before the oxidation of the Mn_4_CaO_5_ cluster in the S_2_ to S_3_ transition (26–28). The difference between the preexisting and postforming low-barrier H-bonds is likely to be associated with the difference in the rate-limiting step (i.e. rate-limiting electron transfer in the S_0_ to S_1_ transition and rate-limiting proton transfer in the S_2_ to S_3_ transition (32)).

A low-barrier H-bond is also present in the electron acceptor TyrZ…D1-His190 pair (33, 34). When it (TyrZ-OH) is oxidized to the neutral radical form (TyrZ-O^•^) by P_D1_^•+^, it serves as an electron acceptor for the Mn_4_CaO_5_ cluster. The S_2_ to S_3_ transition is initiated by the formation of TyrZ-O^•^ in S_2_, as confirmed by the detection of the S_2_TyrZ-O^•^ state in electron paramagnetic resonance (EPR) studies upon the inhibition of the S_2_ to S_3_ transition (35, 36). D1-Asp170 at Mn4 provides an electron transfer route between the Mn_4_CaO_5_ cluster and TyrZ-O^•^ (37). In contrast, D1-Glu189 at Mn1 does not provide an electron transfer route to TyrZ-O^•^ (37). The absence of a distinct electron transfer route between Mn1 and TyrZ-O^•^ suggests that the oxidation of Mn1(III) to Mn(IV) occurs less readily than the oxidation of Mn4(III) to Mn4(IV), which aligns with the absence of the closed-cubane conformation with Mn1(IV) in the XFEL structures (12–16). Although the functional link between the formation of TyrZ-O^•^ and the release of the electron from the Mn_4_CaO_5_ cluster has been reported (37), it remains unclear whether a similar link exists between the formation of TyrZ-O^•^ and the release of the proton from the Mn_4_CaO_5_ cluster. In particular, the mechanism of the S_2_ to S_3_ transition, which proceeds via two steps with distinct KIE values, is not fully understood. In this context, QM/MM calculations can explore proton transfers via H-bonds and electron transfers via redox-active groups simultaneously in the protein environment, providing detailed insights into the complex interplay in proton-coupled electron transfer processes, including the formation and loss of covalent bonds and radical species. In this study, we investigated the mechanism of how TyrZ-O^•^ drives proton-coupled electron transfer during the S_2_ to S_3_ transition, using a QM/MM approach and considering TyrZ-O^•^, the Mn_4_CaO_5_ cluster, and the proceeding H-bond network quantumchemically.

## Results

**Fig. 1.**
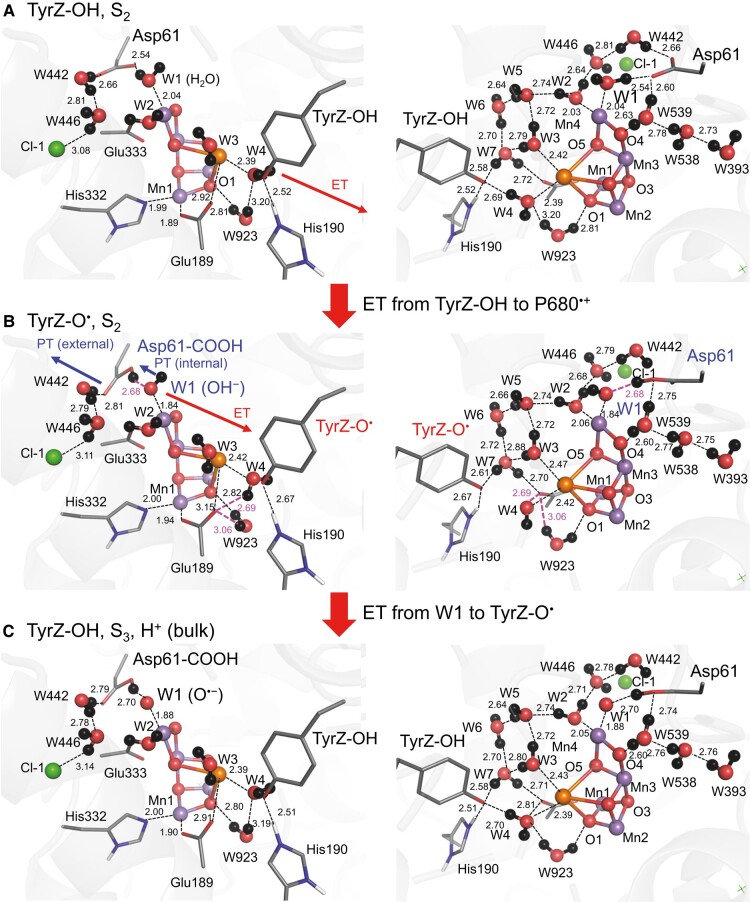
Changes in the QM/MM-optimized geometry in response to formation of TyrZ-O^•^ in S_2_. A) TyrZ-OH in S_2_: before electron transfer from TyrZ-OH to [P_D1_P_D2_]^•+^. B) TyrZ-O^•^ in S_2_: after electron transfer from TyrZ-OH to [P_D1_P_D2_]^•+^. C) TyrZ-OH in S_3_ (or S_3_ precursor): after electron transfer from S_2_ to TyrZ-O^•^. The H-bond pattern for TyrZ is the same as that shown in A). Red and blue arrows indicate electron and proton transfers, respectively. Black dotted lines indicate H-bonds. Pink dotted lines indicate newly formed key H-bonds in response to TyrZ-O^•^ formation. Note that the proton of TyrZ-OH does not necessarily belong to the TyrZ moiety, as it forms a low-barrier H-bond with D1-His190.

The TyrZ…D1-His190 pair forms a short low-barrier H-bond (2.52 Å) in S_2_ before electron transfer occurs, as suggested previously (34) (Fig. 1A). Upon electron transfer from TyrZ to P_D1_^•+^, TyrZ-O^•^ forms and breaks the short low-barrier H-bond with D1-His190 (38). Remarkably, our QM/MM calculation indicates that the formation of OH^–^ at W1 and the protonation of D1-Asp61 via intra H-bond proton transfer already occur upon TyrZ-O^•^ formation (Fig. 1B). In contrast to the predictions of Allgöwer et al. (30) based on density functional theory (DFT) models, deprotonation of W3 at Ca^2+^ is not observed in the present QM/MM calculations, despite W3 being only 3.6 Å away from TyrZ (1). The absence of W3 deprotonation in the present study is consistent with (i) their reported high-energy barrier (11 kcal/mol) for proton transfer from H_2_O at W3 to even (predeprotonated) OH^–^ at W2 (30), with an energy profile distinct from typical low-barrier H-bonds (39). It is important to note that the release of the proton from W3 toward W2 can occur more easily when OH^–^, rather than H_2_O, is assumed on the proton acceptor W2 site. Thus, their reported high-energy barrier even with OH^–^ at W2 demonstrates that the release of the proton from W3 is unlikely to occur. Furthermore, the model assuming OH^–^ at W2 needs reconsideration, as results from Fourier transform infrared spectroscopy (FTIR) (40) and recent pulsed electron–electron double resonance (PELDOR) (41) studies support W2 being H_2_O in S_2_. While their proposed W3 deprotonation model might be useful for reasoning the mechanism of the incorporation of an additional water molecule (e.g. O6 (12, 13)) into the Mn_4_CaO_5_ cluster, the high-energy barrier for proton release, even when considering dynamics, implies that an alternative mechanism is more relevant to this process. The present finding indicates that deprotonation of W1 can occur readily without the contribution of dynamics (e.g. KIE = 1.2 (19)) prior to deprotonation of W3.

**Fig. 2.**
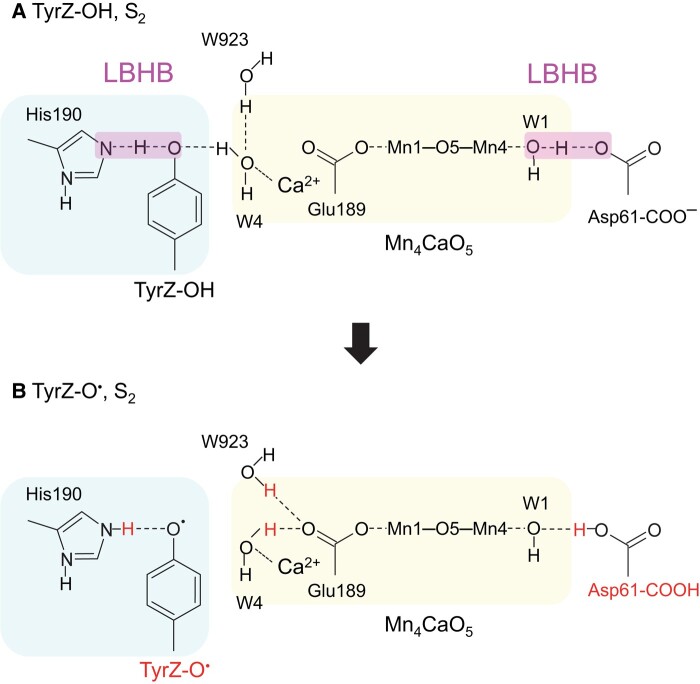
Changes in the H-bond pattern and loss of low-barrier H-bonds (LBHBs) at the Mn_4_CaO_5_ interfaces in S_2_. A) TyrZ-OH. B) TyrZ-O^•^. Pink bands indicate LBHBs. Blue and yellow boxes indicate the TyrZ and Mn_4_CaO_5_ moieties.

In the present QM/MM calculations, instead of W3, another ligand water molecule, W4, at Ca^2+^ exhibits a distinct reorientation in the process (see below). Deprotonation of W1 (protonation of D1-Asp61) caused by the *apparent* electrostatic interaction between TyrZ and W1 (D1-Asp61) over a distance of 9 Å (11 Å) is remarkable. Before TyrZ-O^•^ formation, TyrZ accepts an H-bond from W4 at Ca^2+^, and W4 accepts an H-bond from the adjacent water molecule (W923), forming an H-bond network [TyrZ…W4…W923] (Figs. 1A and 2A). Upon TyrZ-O^•^ formation, W4 and W923 cannot form the H-bond with TyrZ-O^•^, thereby both reorienting and each donating an H-bond to negatively charged D1-Glu189 at Mn1 of the Mn_4_CaO_5_ cluster, respectively (Figs. 1B and 2B). Thus, in response to the formation of TyrZ-O^•^, the Mn_4_CaO_5_ cluster additionally accepts two H-bonds, releasing the proton from W1 toward D1-Asp61 along the preexisting low-barrier H-bond (Figs. 1B and 2B). The release of the proton from W1 toward D1-Asp61 is consistent with QM/MM/MD calculations by Narzi et al. (24).

**Fig. 3.**
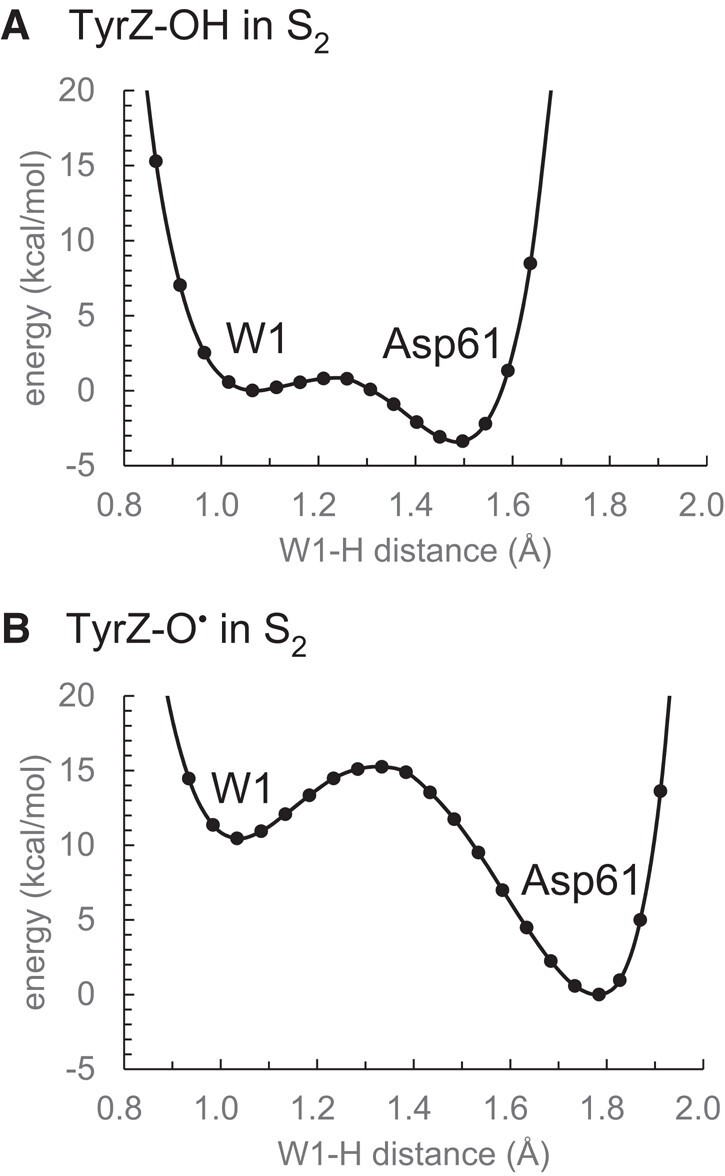
Changes in the energetics of the H-bond between H_2_O at W1 and D1-Asp61 in response to the formation of TyrZ-O^•^ in S_2_: A) in the presence of TyrZ-OH and B) in the presence of TyrZ-O^•^.

The QM/MM calculation results in the formation of TyrZ-O^•^, OH^–^ at W1, and protonated D1-Asp61, as long as the TyrZ…D1-His190 pair is included in the QM region (Fig. 1). To assess how alterations in the H-bond arrangement at the interface of TyrZ and the Mn_4_CaO_5_ cluster impact the release of the proton from W1 toward D1-Asp61, the potential energy profile of the H-bond between W1 and D1-Asp61 is analyzed using the QM/MM-optimized geometries for TyrZ-OH (Fig. 1A) and TyrZ-O^•^ (Fig. 1B) but excluding the TyrZ…D1-His190 pair from the (quantumchemically treated) QM region and assigning the TyrZ-O^•^ charge. The potential energy profile of the H-bond between W1 and D1-Asp61 indicates that W1 forms a low-barrier H-bond with D1-Asp61 in the presence of TyrZ-OH (Fig. 3A). However, in response to the formation of TyrZ-O^•^, proton transfer from W1 to D1-Asp61 becomes energetically downhill (Fig. 3B). As the potential energy profile is analyzed excluding the TyrZ…D1-His190 pair from the QM region, the difference in the potential energy profile between TyrZ-OH and TyrZ-O^•^ solely originates from the difference in the geometry, namely, the H-bond pattern near TyrZ. The result suggests that TyrZ-O^•^ does not directly interact with D1-Asp61 at a distance of 11.4 Å electrostatically: it only interacts with the Mn_4_CaO_5_ cluster via D1-Glu189 at a distance of only 3.9 Å (Fig. S1) via the H-bond pattern change (Fig. 2). The rearrangement of the H-bonds induced by TyrZ-O^•^, not the increase of the net charge (i.e. from TyrZ-OH…N-His190 to TyrZ-O^•^ [HN-His190]^+^), plays a major role in decreasing the p*K*_a_ value and releasing the proton from the W1 site to the H-bond acceptor, D1-Asp61 (Table S1).

**Fig. 4.**
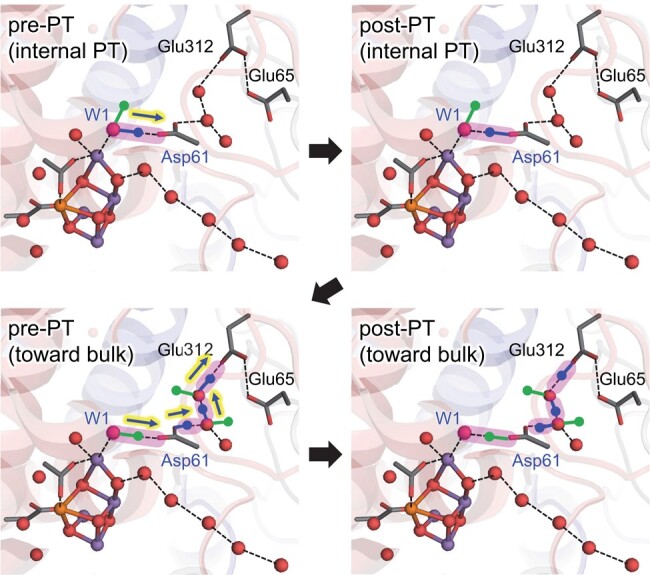
H-bond patterns for pre-PT and post-PT H-bond network during internal proton transfer for protonation of D1-Asp61, formation of the Grotthuss-like H-bond network, and release of the W1 proton along the D1-Glu65/D2-Glu312 channel toward the protein bulk surface. Dotted lines represent H-bonds between O atoms or between O and H atoms if H atoms are shown. Blue and green lines and balls indicate protons of the O sites. Pink bands indicate proton-conducting H-bonds. Note that only a representative H-bond network for proton transfer is shown for clarity (29).

The downhill proton transfer sufficiently stabilizes the released proton at the D1-Asp61 moiety. Once protonated, the protonated O site of D1-Asp61 frequently orients toward the D1-Glu65/D2-Glu312 channel (42) and forms an H-bond network with water molecules during MD simulations (26, 29) (Fig. 4). Thus, OH^–^ at W1 donates an H-bond to the unprotonated O site of D1-Asp61, and the protonated O site of D1-Asp61 provides an H-bond to a water molecule located in the D1-Glu65/D2-Glu312 channel (W507 (1)), forming a Grotthuss-like H-bond network.

QM/MM calculations suggested that the proton at the protonated O site of D1-Asp61 can be released along the H-bond network toward D1-Glu65/D2-Glu312 (29). The release of the proton from the protonated O site of D1-Asp61 is synchronized with the release of the proton from OH^–^ at W1 due to the formation of the Grotthuss-like H-bond network that bridges W1 and D1-Glu65/D1-Glu312 (Fig. 4). The release of the proton from W1 is also coupled with electron transfer to TyrZ-O^•^. Eventually, TyrZ-O^•^ is reduced back to TyrZ-OH, with D1-His190 serving as the proton donor, and OH^–^ at W1 in the Mn_4_CaO_5_ cluster is further oxidized to O^•–^ during proton-coupled electron transfer (Fig. 1C). The corresponding proton-coupled electron transfer was also reported for O_2_ evolution mediated by carboxylic acid on the α-MnO_2_ electrode (43).

It should be noted that the entire proton transfer in the S_2_ to S_3_ transition is a 1 H^+^ process, because the proton of H_2_O at W1 is already delocalized along the low-barrier H-bond with D1-Asp61 before TyrZ-O^•^ formation, and the increase in the p*K*_a_ value of W1 can mostly be used as a driving force for 1 H^+^ proton transfer toward the lumenal bulk region in the second step (Figs. 1 and 4).

The observed two-step proton transfer, the first internal proton transfer followed by the second proton-coupled electron transfer, fits the kinetics estimated using photothermal beam deflection by Klauss et al. (21, 22) and using TRIR spectroscopy by Shimizu et al. (19). The first step with KIE = 1.2 was as low as that for the S_0_ to S_1_ transition (19), which fits proton transfer along the preexisting low-barrier H-bond presented in this study.

KIE = 1.5 was reported for proton-coupled electron transfer in O_2_ evolution mediated by carboxylic acid on the α-MnO_2_ electrode (43), which resembles proton-coupled electron transfer via D1-Asp61 in PSII. Given that the formation of the proton-conducting wire is an activation step for proton transfer (20), it seems plausible that the second proton-coupled electron transfer, which involves the formation of the Grotthuss-like H-bond network extending from W1 toward D1-Glu65/D2-Glu312, leads to a large KIE value with respect to the first internal proton transfer (Fig. 4).

The mechanism presented in this study does not necessarily exclude the incorporation of O6 into the O5 moiety during the S_2_ to S_3_ transition (12–16). Although it is not specifically investigated in the present study, the process may follow the proton-coupled electron transfer observed in the present study.

## Discussion

### Comparison with previously proposed mechanisms for protonation of D1-Asp61

**Fig. 5.**
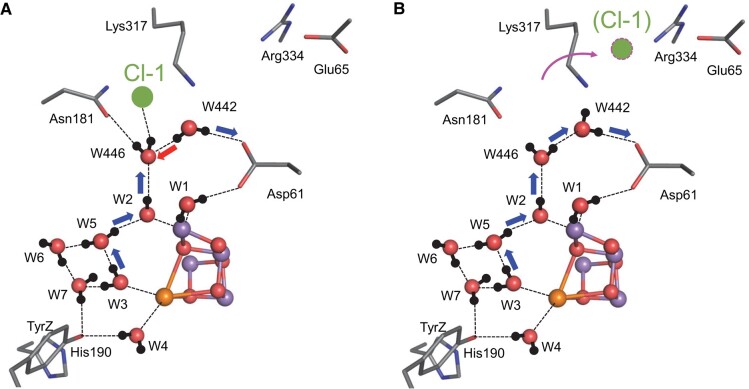
Schematic view of the H-bond network at the Cl-1 binding moiety in the presence of OH^–^ at W2 proposed by Allgöwer et al. (30). A) Cl-1 at the D1-Asn181 and D2-Lys317 sites, as originally identified in the PSII structures. B) Displacement of Cl-1 toward D1-Arg334 proposed by Allgöwer et al. (30). As the atomic coordinates are not provided in Allgöwer et al. (30), the geometry shown was generated based on the PSII structure. Dotted lines indicate H-bonds. Blue arrows indicate the H-bond donor to acceptor orientations. The red arrow indicates the H-bond that inhibits the formation of a Grotthuss-like proton-conducting H-bond network between W3 and D1-Asp61. See Fig. 1 for the H-bond network in the presence of H_2_O at W2.

Theoretical studies by Allgöwer et al. (30) proposed a release of the proton from W3 at the redox-inactive Ca^2+^ site followed by a sequential proton transfer along a series of water molecules (W5, W2, W446, and W442) near Cl-1, ultimately reaching D1-Asp61 in the S_2_ to S_3_ transition (Fig. 5B). If these water molecules function as a proton-conducting H-bond network, they would need to form a Grotthuss-like H-bond network. However, the original PSII structures do not allow them to form it: specifically, the OH group of the water molecule at the Cl-1 binding moiety, W446, orients toward the negatively charged Cl-1 ion instead of orienting toward an anticipated proton acceptor water molecule, W442, disrupting the formation of a Grotthuss-like H-bond network (Fig. 5; see below).

In the model proposed by Allgöwer et al. (30), OH^–^ was placed at W2. However, FTIR (40) and PELDOR (41) studies suggested that W2 corresponds to H_2_O in both S_1_ and S_2_. Additionally, p*K*_a_ calculations performed by Saito et al. (27) revealed that p*K*_a_(W1) was notably lower than p*K*_a_(W2) in the PSII protein environment owing to the presence of the proton acceptor, D1-Asp61. The involvement of H_2_O at W2 in the H-bond network suggests that these water molecules cannot efficiently form a proton-conducting H-bond network, as the ligand O site interacts with Mn4 and thereby cannot accept an H-bond (Fig. 1). Consequently, all theoretical studies, which assume proton transfer via W2, necessitate the presence of OH^–^ at W2 to accept an H-bond from W3 (Fig. 5A) (10, 11, 44–46). However, caution must be taken, as the idea of OH^–^ at W2 was originally postulated based on a simplified computational model (Mn_4_CaO_5_ and the ligand groups), which lacks the PSII protein environment (10). Indeed, recent QM/MM studies conducted in the presence of the PSII protein environment unambiguously suggested that the EPR signals observed in S_2_ cannot be reproduced when W2 = OH^–^ (41, 47).

Even if OH^–^ at W2 is assumed, the original atomic coordinates of the X-ray diffraction (XRD) (1) and S_2_-XFEL structures (12–16) do not allow the formation of the proposed Grotthuss-like H-bond network due to the presence of Cl-1. As long as Cl-1 exists at the binding site, D1-Asn181 and D2-Lys317, one of the H atoms of W446 orients strongly toward anionic Cl-1 and cannot donate an H-bond to W442, interrupting the formation of a stable Grotthuss-like H-bond network (48) (Fig. 5A). Consistently, the mechanism proposed by Allgöwer et al. (30) proposed a high-energy barrier of 13 kcal/mol for the release of the proton from W446 to D1-Asp61 via W442 (W_c121_ (30)).

The proposed mechanism (30) also suggests the displacement of Cl-1 from the original binding site (D1-Asn181 and D2-Lys317) toward the adjacent basic residue, D1-Arg334, during the proton transfer from W3 to D1-Asp61 (Fig. 5B). However, Cl-1 at the D1-Arg334 moiety is not identified in reported native PSII structures (1, 12–16). Furthermore, MD simulations by Rivalta et al. (23) showed that the loss of Cl-1 at the D1-Asn181 and D2-Lys317 moieties leads to the formation of a salt bridge between D1-Asp61 and D2-Lys317, interrupting proton transfer via D1-Asp61 and likely inhibiting the S_2_ to S_3_ transition, in contrast to the mechanism proposed by Allgöwer et al. (30). Similar structural changes were also observed in QM/MM calculations by Mandal et al. (49).

If the movement of Cl-1 between the two binding sites were coupled with the redox change in TyrZ, the proton transfer would have required significant rearrangement of the H-bond network to form a Grotthuss-like H-bond network, leading to less efficient proton transfer with several activation steps (20). Consistently, in the mechanism proposed by Allgöwer et al. (30), the first energy barrier of 11 kcal/mol exists for proton transfer from W3 toward W446 (W_c109_ (30)) via W5 (W_c9_ (30)) and W2 even in the presence of TyrZ-O^•^, and the second energy barrier of 13 kcal/mol exists for the release of the proton from W446 to D1-Asp61 via W442 (W_c121_ (30)). The presence of these two high energy barriers suggests that proton transfer is unlikely to occur along the proposed pathway within the experimentally measured time scale (∼300 μs (19, 21)). In contrast, an alternative mechanism presented in this study suggests that the protonation of D1-Asp61 upon TyrZ-O^•^ formation occurs exothermically (Fig. 2). Consequently, the energy barrier is significantly smaller in the mechanism presented in this study than that proposed by Allgöwer et al. (30). PSII does not necessarily undergo a process with a higher energy barrier, inefficiently deprotonating W3 at the redox-inactive Ca^2+^ site (11 kcal/mol (30)) and inefficiently transferring the proton along a non–Grotthuss-like H-bond network (13 kcal/mol (30)).

If D1-Arg334 served as the second binding site and played a key role in transiently displacing Cl-1 and facilitating the formation of a proton transfer pathway via W446 and W442 upon the formation of TyrZ-O^•^, the mutations of positively charged D1-Arg334 would have inhibited the S_2_ to S_3_ transition. However, the mutations of D1-Arg334 to the other 19 residues had only a minor impact on their photosynthetic growth (29). In particular, the mutation of D1-Arg334 to hydrophobic residues did not inhibit their photosynthetic growth (29), which suggests that the involvement of D1-Arg334 in serving as an alternative Cl-1 binding site to facilitate the formation of the proton-conducting wire via W446 and W442 is less likely. Furthermore, the displacement of Cl-1 from the D1-Asn181 and D2-Lys317 moieties toward the D1-Arg334 was also observed in the *Synechocystis* PSII structure lacking membrane-extrinsic protein subunits and the Mn_4_CaO_5_ cluster (apo PSII) (50). D1-Arg334 orients toward and interacts with Cl-1 in the apo PSII structure, as observed in the S_2_ geometry presented by Allgöwer et al. (30). Thus, the displacement of Cl-1 from the original binding site toward D1-Arg334 (30) may be in line with the inactive apo PSII structure (50) rather than the native PSII structure (1).

### Roles of the cluster components

Changes in the H-bond pattern at the D1-Glu189 moiety of the Mn_4_CaO_5_ cluster upon the formation of TyrZ-O^•^ are observed predominantly at W4 but not at W3 (Fig. 1). Remarkably, the PSII crystal structures show that upon replacement of Ca^2+^ with Sr^2+^, the [Ca^2+^/Sr^2+^]…W4 distance remains unchanged, but the [Ca^2+^/Sr^2+^]…W3 distance is lengthened (51). The similar [Ca^2+^/Sr^2+^]…W4 distances may explain why Sr^2+^ substitution does not inhibit evolution (52–56), as proton transfer is not inhibited according to the mechanism presented in this study.

**Fig. 6.**
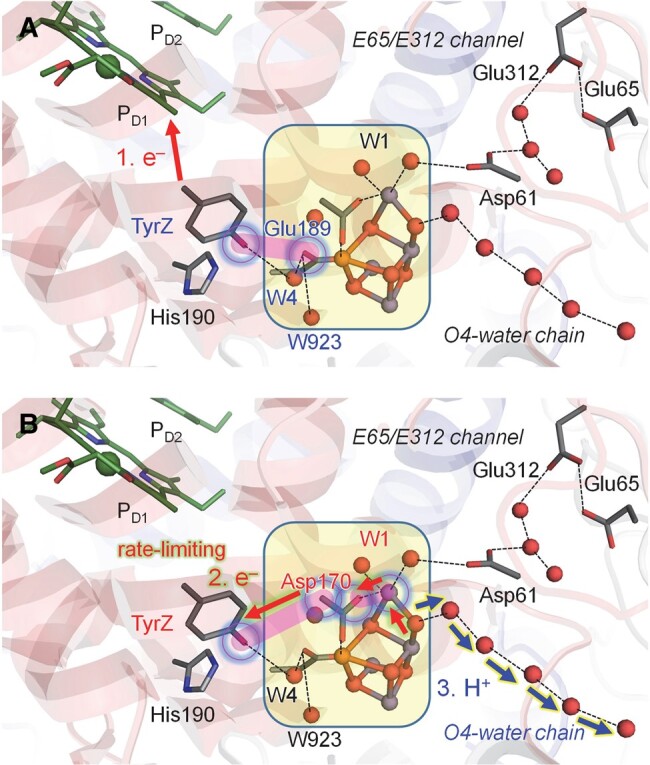
Reaction interface between TyrZ and the Mn_4_CaO_5_ cluster in the S_0_ to S_1_ transition. A) Electron transfer from TyrZ-OH to P_D1_^•+^. B) Electron transfer via the D1-Asp170…TyrZ interface and proton transfer toward the protein bulk surface. The boxed square indicates the Mn_4_CaO_5_ cluster, including the ligand groups. Blue open circles indicate active sites. Pink bands indicate interactions between active sites. Note that electron transfer is rate limiting (32), occurring from Mn3 via Mn4 and D1-Asp170 transition (37) in the S_0_ to S_1_ transition.

Based on these observations, Ca^2+^ may play a role in serving as a flexible binding site for W4 adjacent to TyrZ, transmitting the signal (TyrZ-O^•^ formation) to the Mn_4_CaO_5_ cluster via D1-Asp189. D1-Glu189 at Mn1 plays a role in sensing radical formation via W4, decreasing the p*K*_a_ of the Mn_4_CaO_5_ cluster and eventually facilitating the release of the proton from W1 (Fig. 6).

The present result suggests that D1-Glu189 provides a direct link between TyrZ-O^•^ formation and Mn_4_CaO_5_ deprotonation. The role of D1-Glu189 at Mn1 in proton transfer from the Mn_4_CaO_5_ cluster is comparable with that of D1-Asp170 at Mn4 in electron transfer from the Mn_4_CaO_5_ cluster (37). It has been postulated that D1-Asp170 and D1-Glu189 are involved in the photoassembly process of the Mn_4_CaO_5_ cluster. They serve as the initial binding sites for Mn^2+^ and facilitate the oxidation of Mn^2+^ to Mn^3+^ (57, 58). Moreover, recent investigations by Shimada et al. (59) have demonstrated that, even when these two residues are mutated, they undergo posttranslational modifications, reverting to their original forms as D1-Asp170 and D1-Glu189. Based on the present results, the unique characteristics are likely due to their essential roles in electron transfer and proton transfer via TyrZ-O^•^.

### Comparison with the release of the proton in the S_0_ to S_1_ transition

**Fig. 7.**
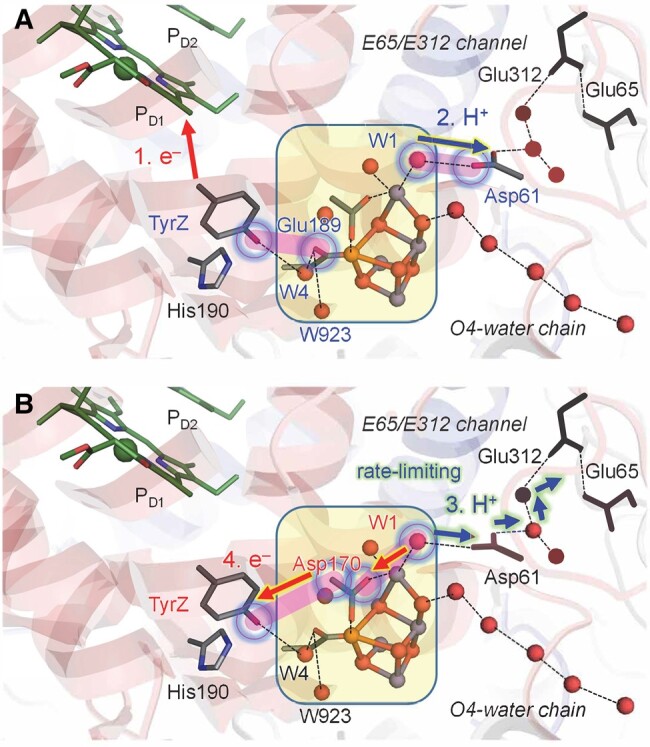
Reaction interface between TyrZ and the Mn_4_CaO_5_ cluster in the S_2_ to S_3_ transition. A) Electron transfer from TyrZ-OH to P_D1_^•+^ and proton transfer from W1 to D1-Asp61 triggered via the TyrZ…D1-Glu189 interface. B) Electron transfer via the D1-Asp170…TyrZ interface and proton transfer toward the protein bulk surface. The boxed square indicates the Mn_4_CaO_5_ cluster, including the ligand groups. Blue open circles indicate active sites. Pink bands indicate interactions between active sites. Note that proton transfer is rate limiting in the S_2_ to S_3_ transition (32).

As no significant structural difference was observed at the TyrZ…Mn_4_CaO_5_ moiety among the XRD (1), S_1_-XFEL, and S_2_-XFEL structures (12–16), it seems highly likely that the H-bond rearrangement in the TyrZ, W4, W923, and D1-Glu189 region observed in the S_2_ to S_3_ transition may also occur in the S_0_ to S_1_ transition (Fig. 6). The proton-releasing site, W1, already forms a low-barrier H-bond with D1-Asp61 in S_2_ (26–28) (due to the absence of the release of the proton in the S_1_ to S_2_ transition), whereas the proton-releasing site, O4, does not form a low-barrier H-bond with the O4–water chain in S_0_ (17, 18). Only after electron transfer occurs from the Mn_4_CaO_5_ cluster to TyrZ-O^•^, O4 does form a low-barrier H-bond with the O4–water chain, facilitating proton transfer (17, 18). This discrepancy is likely responsible for the difference in the rate-limiting step for the proton-coupled electron transfer between the S_0_ to S_1_ (rate-limiting electron transfer) and S_2_ to S_3_ (rate-limiting proton transfer) transitions (Figs. 6 and 7).

## Conclusion

In S_2_, the formation of TyrZ-O^•^ leads to the loss of the low-barrier H-bond between TyrZ and D1-His190 (33) and the loss of the H-bond between TyrZ and W4 (Fig. 1B). Eventually, the Mn_4_CaO_5_ cluster accepts H-bonds via D1-Glu189 at the Mn1 moiety, decreasing the p*K*_a_ value, namely, for the lowest p*K*_a_ site, W1 (Figs. 2 and 3; Table S1). Internal proton transfer occurs in the preexisting low-barrier H-bond between W1 and D1-Asp61 and may correspond to the KIE = 1.2 process (19). The reorientation of the protonated O site of D1-Asp61 toward the D1-Glu65/D2-Glu312 channel leads to the formation of a Grotthuss-like H-bond network that proceeds from W1 via D1-Asp61 toward D1-Glu65/D2-Glu312 (Fig. 4). Electron transfer occurs from W1 to TyrZ-O^•^ via the ligation site Mn4 and the electron-transfer route, D1-Asp170 (37). As the reorientation of the D1-Asp61 side chain, formation of the H-bond network, and the proton-coupled electron transfer at the second step require greater rearrangements of the protein environment than at the first step, the second step may correspond to the KIE = 1.9 process (19). Thus, TyrZ-O^•^ induces the protonation of D1-Asp61 (TyrZ…D1-Asp61 = 11.4 Å; Fig. S1), only changing the H-bond pattern (Fig. 2) and interacting with D1-Glu189 (TyrZ…D1-Glu189 = 3.9 Å; Fig. S1) of the Mn_4_CaO_5_ cluster (Fig. 7). Not the direct electrostatic interaction between TyrZ-O^•^ and D1-Asp61 (30) but the H-bond rearrangement along the extended Grotthuss-like network [TyrZ…D1-His190]-[Mn_4_CaO_5_]-[W1…D1-Asp61] provides the main driving force for protonation of D1-Asp61 (Table S1). It seems likely that D1-Glu189 at Mn1 facilitates proton transfer by sensing TyrZ-O^•^ formation, whereas D1-Asp170 facilitates electron transfer by providing an electron transfer route (37) to TyrZ-O^•^ (Fig. 7). Deprotonation of W3 is not observed upon TyrZ-O^•^ formation (Fig. 1B). This is because (i) TyrZ forms an H-bond with W4 (2.9 Å) but not with W3 (3.6 Å), (ii) Ca^2+^ is a weak Lewis acid (60), and (iii) the energy barrier is high (11 kcal/mol) (30).

While the mechanisms of efficient proton transfer, in which the preorganized proton-conducting wire is a prerequisite, are universal (20), the mechanism presented in this study possesses unique features. (i) The proton-conducting wire involves two low-barrier H-bonds (TyrZ…D1-His190 (33, 34, 61) and W1…D1-Asp61 (26)) and (ii) a metal complex, the Mn_4_CaO_5_ cluster, also mediates the proton-coupled electron transfer as a component of the H-bond network (Fig. 2). The interplay of two low-barrier H-bonds, involving the Ca^2+^ interface and forming the extended Grotthuss-like network [TyrZ…D1-His190]-[Mn_4_CaO_5_]-[W1…D1-Asp61], enables the long-distance proton-coupled electron transfer over 10 Å, involving radical species. The proton remains delocalized between TyrZ and D1-His190 during the S-state transitions, whereas the proton is released away from D1-Asp61 toward the protein bulk surface in the S_2_ to S_3_ transition. This difference makes the network specifically effective during the S_2_ to S_3_ transition. The involvement of the Ca^2+^ interface in the [TyrZ…D1-His190]-[Mn_4_CaO_5_]-[W1…D1-Asp61] network may elucidate the specific requirement of Ca^2+^ for the S_2_ to S_3_ transition, given that D1-Asp61 forms a low-barrier H-bond during this S-state transition. These findings may serve as a molecular basis for possible proceeding events, e.g. incorporation of a substrate water molecule into the Mn_4_CaO_5_ cluster.

## Methods

The atomic coordinates were obtained from the XRD structure of the PSII monomer unit “A” in *Thermosynechococcus vulcanus* PSII complex (PDB code, 3ARC) (1). Crystallographically resolved water molecules were explicitly included, with no additional water molecules introduced, as those adjacent to the Mn_4_CaO_5_ cluster are expected to exhibit minimal disorder, as indicated by the XRD structure (1). Atomic partial charges of the amino acids and cofactors were derived from the CHARMM22 (62) parameter set and previous studies (17), respectively. D1-His337 was treated as protonated (40), with all other titratable groups treated to be ionized.

The B3LYP functional and LACVP* basis sets were utilized in the unrestricted DFT method (LANL2DZ for Mn and Ca; 6–31G* for other atoms) (63) in the QSite program (64). To maintain charge neutrality, counter ions were introduced. In the QM region, default geometry optimization algorithms were employed in QSite, and all atomic coordinates were relaxed with the default convergence criteria (47). The van der Waals parameters of the OPLS2015 force field were employed (65). In the MM region, the positions of H atoms were optimized energetically, while the positions of heavy atoms remain fixed. This approach ensured that the MM region primarily replicated electrostatic interactions with the QM region, while the positions of heavy atoms remained consistent with those in the original crystal structure, preventing unrealistic displacement of heavy atoms in the MM region as artifacts. The initial-guess wavefunctions were generated using ligand field theory (66). The QM region encompassed several components, including the Mn_4_CaO_5_ cluster (comprising Mn_4_CaO_5_, the ligand side-chains; the carboxyl-terminal group of D1-Ala344; and W1–W4), O4–water chain (W539, W538, and W393) (17, 18), Cl-1 binding site (Cl-1, W442, W446, and D1-Asn181 and D2-Lys317 side chains), second-sphere ligands (side chains of D1-Asp61 and CP43-Arg357), and the H-bond network of TyrZ (D1-Tyr161, D1-His190, and D1-Asn298 side chains, W5, W6, and W7) (33, 34). Detailed atomic coordinates of the QM/MM-optimized geometry are provided in the supplementary material.

The initial geometry for analyzing the potential energy profiles of the H-bond (e.g. O…H^+^ …O) was based on the QM/MM-optimized geometry. To investigate the effect of altering the H-bond geometry during the process of TyrZ-O^•^ formation, the QM region was redefined to include the Mn_4_CaO_5_ cluster, O4–water chain (W539) (17, 18), Cl-1 binding site (Cl-1 and W442), and second-sphere ligands. A focusing H atom was systematically transferred along the O…H^+^…O bond by 0.05 Å. After each shift, the geometry was optimized while maintaining the fixed O…H and H…O distances, and the corresponding energy was calculated. This iterative process continued until the H atom reached the proton donor or acceptor O moieties.

## Supplementary Material

pgad423_Supplementary_Data

## Data Availability

All data are included in the manuscript and/or supplementary material.
