# Supplementary material for: Interplay of two low-barrier hydrogen bonds in long-distance proton-coupled electron transfer for water oxidation
Source: PNAS Nexus. 2023 Dec 7;2(12):pgad423. doi: 10.1093/pnasnexus/pgad423 (PMC10733176; doi:10.1093/pnasnexus/pgad423)
Supplement: pgad423_Supplementary_Data [file pgad423_supplementary_data.zip › PNASNEXUS-PNASNEXUS-2023-00889R-s02.pdf]

Supporting Information

Interplay of two low-barrier hydrogen bonds in long-distance proton-coupled electron transfer for water oxidation

Keisuke Saito [1,2]*, Shunya Nishio [1], and Hiroshi Ishikita [1,2]*

1) Department of Applied Chemistry, The University of Tokyo, 7-3-1 Hongo, Bunkyo-ku, Tokyo 113-8654, Japan

2) Research Center for Advanced Science and Technology, The University of Tokyo, 4-6-1 Komaba, Meguro-ku, Tokyo 153-8904, Japan

CORRESPONDING AUTHOR:

Keisuke Saito, **E-mail:** ksaito@appchem.t.u-tokyo.ac.jp

Hiroshi Ishikita, **E-mail:** hiro@appchem.t.u-tokyo.ac.jp

Research Center for Advanced Science and Technology, The University of Tokyo, 4-6-1 Komaba, Meguro-ku, Tokyo 153-8904, Japan, Tel. +81-3-5452-5056, Fax. +81-3-5452-5083

Table S1. Contributions of the changes in the TyrZ-OH/TyrZ-O$^{\bullet}$ charge and the H-bond pattern to the driving force for proton transfer from W1 to D1-Asp61 (ΔE(PT)) upon formation of TyrZ-O$^{\bullet}$ in Figure 3b (kcal/mol). Contributions were calculated excluding TyrZ from the QM region and replacing the atomic charges of TyrZ.

**ΔE(PT)**	**-10.4**
contributions	
change in the TyrZ-OH/TyrZ-O$^{\bullet}$ charge	-3.5
change in the H-bond pattern	-6.9

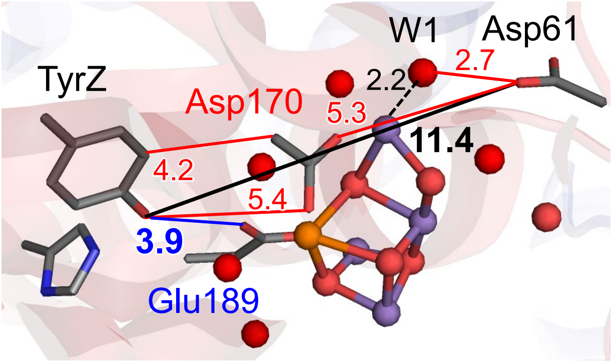

Figure S1. Distances between TyrZ and D1-Asp61 in the PSII crystal structure (Å). Red and blue lines indicate distances with D1-Asp170 and D1-Glu189, respectively.
